# CEA as a risk factor in predicting ocular metastasis from colorectal cancer

**DOI:** 10.7150/jca.31196

**Published:** 2020-01-01

**Authors:** You-Lan Min, Ying-Xin Gong, Pei-Wen Zhu, Qi Lin, Biao Li, Wen-Qing Shi, Qing Yuan, Yi Shao

**Affiliations:** Department of Ophthalmology, The First Affiliated Hospital of Nanchang University, Jiangxi Province Ocular Disease Clinical Research Center, Nanchang 330006, Jiangxi, People's Republic of China

**Keywords:** carcinoma embryonic antigen, ocular metastasis, colorectal cancer, independent risk factor

## Abstract

Objective: Colorectal, closely following pulmonary and breast, is the third predilection site of cancer that lead to death all over the world. Ocular metastasis (OM) of colorectal cancer (CRC) is becoming increasingly common and presents a poor prognosis. In this study, we detected some recognized tumor biomarkers and tried to differentiate the discrepancy between CRC patients with and without OM in order to clarify the risk factor for OM in patients with colorectal cancer. Methods: 1735 patients with colorectal cancer in total from August 2005 to August 2017 were involved in this study. Nonparametric rank sum test and Chi-square test were applied to prescribe whether there were significant differences between OM group and non-ocular metastasis (NOM) group. And binary logistic regression analysis was used to determine the risk factor. Then, we used receiver operating curve (ROC) to assess the diagnostic value of OM in CRC patients. Results: The incidence of OM in CRC patients was 1.12%. No significant differences were found in gender, age, histopathological type, tumor classification and tumor differentiation between OM group and NOM group. Nonparametric rank sum test approved that OM group had higher serum CEA level compared with NOM group. Binary logistic regression indicated that CEA was a risk factor for OM in colorectal cancer patients (p<0.001). ROC curve showed that AUC of CEA was 0.877. The cutoff value of CEA was 12.45 ng/ml, whose sensitivity is 1.000 and its specificity is 0.877. Conclusion: Based on our study, CEA was a risk factor of ocular metastasis in colorectal cancer patients.

## Introduction

Colorectal cancer (CRC) is the leading cause of morbidity and mortality in many countries and the third most common cancer in America 1,2. There is sufficient evidence that the incidence of CRC is tightly associated with diet and physical activity 3. And owing to the character of lifestyle, the incidence of CRC in Asia is increasingly high 4.

The metastasis of colorectal cancer is common among patients, the most familiar regions of metastases from which include liver (77%), peritoneum (25%), and lungs (22%) 5. Until recently ocular metastasis (OM) has been considered rare in colorectal cancer; however, it is becoming increasingly common 6. Moreover, OM could bring a series of clinical symptoms such as eye pain, peculiar sense, blurred vision, and visual field defect, severely affected the quality of patients' life 7. As those ocular symptoms often occur in advanced stage of metastasis, it is vital to find a new method to predict its occurrence.

Currently, several methods were applied to diagnose CRC, including stool tests that preliminary screen for cancer; CT, colonography, and double-contrast barium enema as image examination for further detection, which can help to define precancerous growths and tumor; flexible sigmoidoscopy and colonoscopy for visual inspection 8. However, the majority of these methods are invasive, time-costing and have a lack or limitation of sensitivity. A simple noninvasive sensitive diagnostic method were not only in the interests of CRC patients themselves but would also be beneficiary for doctors 9.

AFP, CEA, CA-199 and CA-125 were common bio-markers of gastrointestinal cancer. The discovery of carcinoma embryonic antigen (CEA) dates back to 1965 when it was first found by Gold and Freedman and recognized as an oncofetal tumor marker 10. It is significant in 70% of cases who had clear diagnosis of colorectal cancer, and becomes the most widely used CRC marker 11. Michelson et al 12 found that three quarters of patients with metastatic ocular tumor demonstrated higher serum CEA levels. However, the correlation between the ocular metastasis in colorectal cancer and the CEA level in serum still remains uncertain.

In this study, we conducted a large population retrospective study to clarify the correlation between serum tumor biomarkers and ocular metastasis in CRC patients, and attempted to clarify the independent risk factors in detecting ocular metastasis.

## Materials and Methods

### Study design

This study was performed under the approval of the medical research ethics committee of the First Affiliated Hospital of Nanchang University. The methods in this study were conducted under relevant guidelines and regulations. A series of consecutive patients involved in this study had clear diagnosis as colorectal cancer in our hospital between August 2005 and August 2017. All participants involved were offered the whole study design and signed the informed consent. Both OM and NOM patients were diagnosed CRC based on the histopathological examination of their tissues which were obtained from surgical resection or biopsy. Secondary colorectal cancer was excluded from the study. The OM diagnosis was identified by CT and MRI. And the exclusion criteria of OM group were: 1) patients with primary ocular malignant tumor; 2) ocular benign tumor; 3) colorectal cancer patients only with other distant metastases (lung, bone, liver, brain et al).

### Data collection

Relevant clinical data of this retrospective study were collected by patient's medical records, including age, gender, histopathologic types, tumor classification, tumor differentiation, metastases sites and accepted serum tumor markers including AFP, CEA, CA-125 and CA-19-9, whose normal range was 0-7 ng/ml, 0-6.5 ng/ml, 0-35 μ/ml, 0-27 μ/ml, respectively. All the clinical parameters were collected at the time when initial diagnosis of colorectal cancer was made.

### Statistical analysis

Nonparametric sum up test and Chi-square test were applied to find out whether there were differences in the clinical features between OM patients and NOM patients. Then binary logistic regression models were built to clarify the independent risk factors of ocular metastasis. In order to estimate the accuracy of the OM prediction, receiver operating characteristics (ROC) curve was made and areas under the curve (AUC) was calculated. P < 0.05 represented statistically significant. All the statistical analysis was performed by SPSS17.0 software (SPSS, IBM Corp, USA), MedCalc18.6.0 statistical software (MedCalc, Ostend, Belgium) and Excel 2010 software (Excel, Microsoft Corp, USA). Continuous data were displayed in a form of means ± standard deviation (SD).

## Results

### Demographics and clinical characteristics

**Figure 1:**
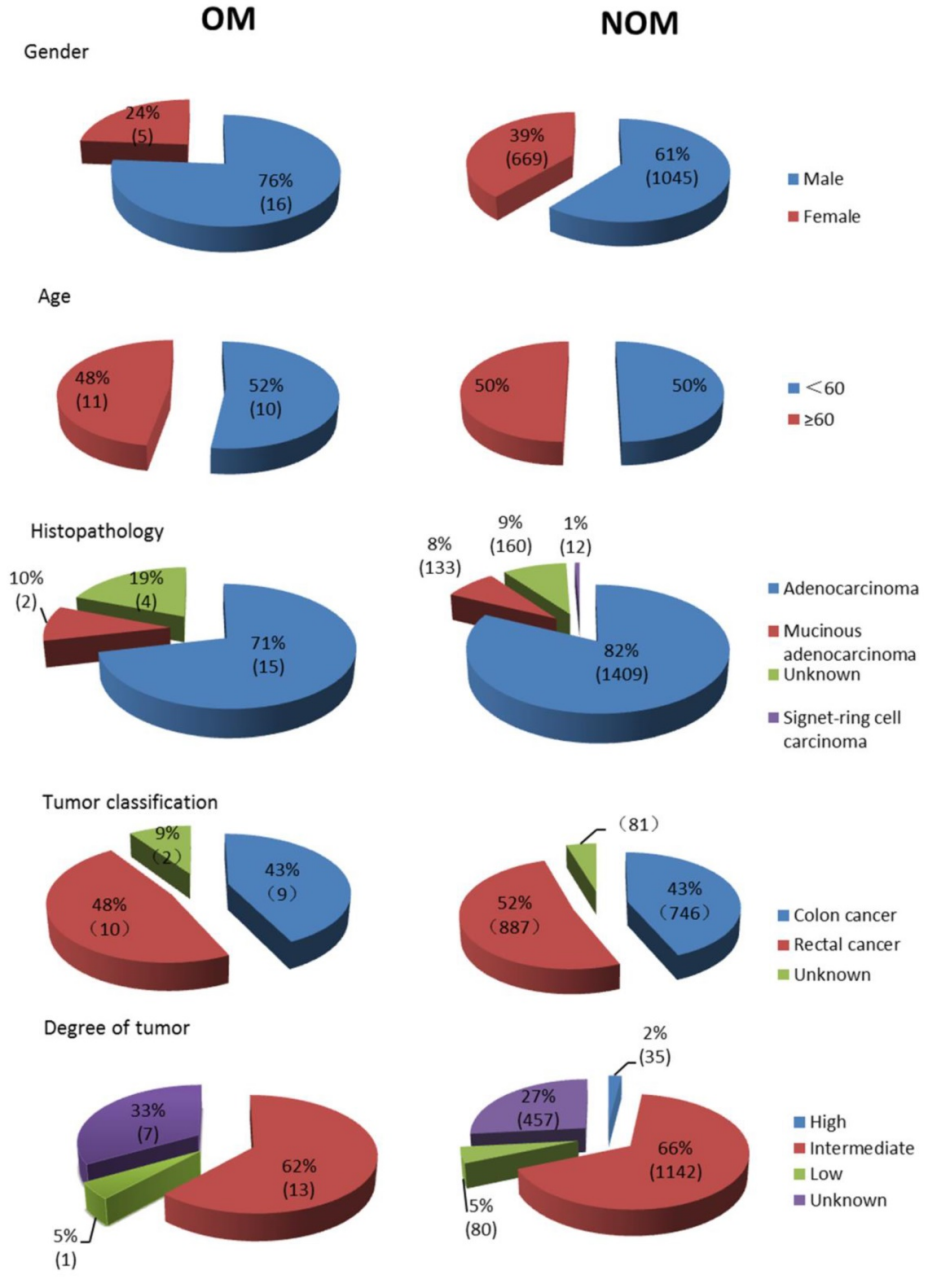
Clinical features of colorectal cancer OM patients and NOM patients. **Notes:** n=21 in OM group, n=1714 in NOM group. OM group included 16 orbital metastasis cases and 5 intraocular metastasis cases. **Abbreviations:** OM, ocular metastasis; NOM, non-ocular metastasis.

**Figure 2:**
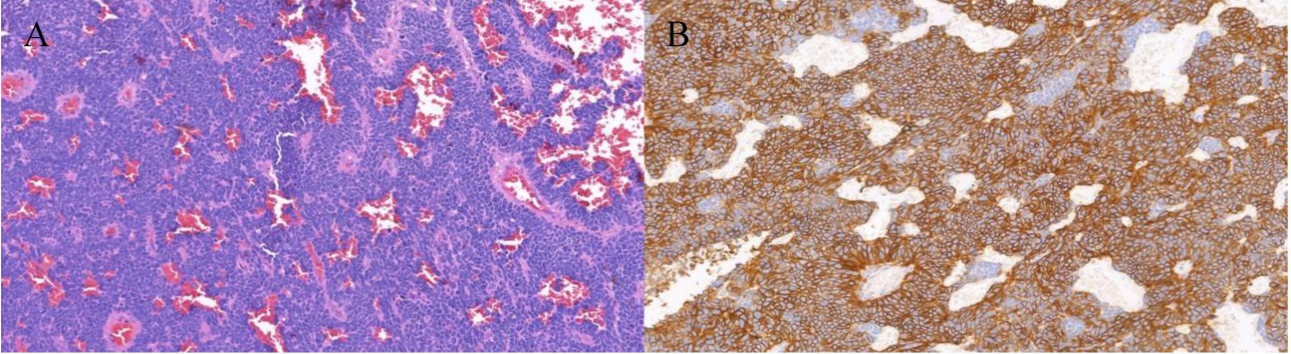
The HE staining and IHC images from colorectal cancer patients with ocular metastasis. **Notes: A.** Colorectal cancer (HE×200) **B.** CD56(+) (SP×200) The tissue was collected from ocular metastasis site of colorectal patients.

A total of 1735 patients (1061 men and 674 women) were recruited in this study, which contained 21 OM cases (16 orbital metastasis cases and 5 intraocular metastasis cases) and 1714 NOM cases. The average age of OM patients and NOM patients were 57.62±12.10 and 57.95±13.36 years old respectively. The majority of the histopathological types were adenocarcinoma (1424 cases, 82.1%), and most of the tumor classifications were rectal cancer (897 cases, 51.7%). As for the degree of tumor differentiation, intermediate differentiation accounted for the largest part (66.6%). According to Chi-square test and nonparametric sum up test there are no significant different (P > 0.05) between OM group and NOM group in gender, age, histopathological type, tumor classification and tumor differentiation. Table 1 also described distant metastasis in both groups. The detailed clinical characteristics of the CRC patients are shown in Table 1 and Figure 1. Figure 2 showed representative HE staining and IHC images of tissue collected from metastasis site (ocular) of colorectal cancer.

### Differences of the clinical features and the risk factors of ocular metastasis

There were no significant differences in the levels of AFP, CA-125, CA-199 between OM patients and NOM patients (P > 0.05). However, higher level of CEA was found in OM patients than NOM patients. The mean level of CEA in OM group was 96.34±168.00 ng/ml, while NOM group was 10.69±49.43 ng/ml. The comparison results were detailed in Table 2. Table 3 analyzed serum CEA between OM patients (n=21) and NOM patients with other organ metastases (n=138), and suggested that the difference of serum CEA concentration was significant.

Binary logistic regression model result showed that CEA could be regarded as independent risk factor of OM. The detailed result was shown in Table 4.

### The cut-of value, AUC, sensitivity and specificity of CEA for diagnosing ocular metastasis

As shown in Figure 2, AUC was 0.943 for CEA, and the sensitivity and specificity were 1.000 and 0.877 respectively. Based on the figure, the cut-off value were 12.45 ng/ml for CEA. All the results were statistically significant.

## Discussion

Colorectal cancer is one of the most common cancers, accounting for about 8.5% of all cancer deceases and 10% of new cases 13. And the metastasis of CRC was considered as the major cause of death, which still remains as a tough problem for early-detection. Approximately 20% of CRC patients already had metastasis when the diagnosis was first made. In addition, 35-45% of the non-metastatic patients succumb to recurrence within 5 years after surgery, and most of these relapses originated in undiscovered preoperative metastases 14. The study suggests that the early diagnosis of metastasis of CRC is lack of enough sensitivity.

The incident of ocular metastasis of CRC is rare comparing to the liver and lungs 5, but now it is becoming more and more common. In 2004, Linares et al 15 described a 47-year-old male with bilateral choroidal metastasis as the initial manifestation of a rectal cancer. In 2008, Kuo et al 16 reported a 65-year-old woman with subfoveal choroidal metastasis from colorectal adenocarcinoma. Moreover, OM represented a poor prognosis signal and could be seen in various kinds of cancers. According to the survey made by Carol et al 17 early in 1997, among all 420 patients recruited, the number of patients developing ocular metastasis from an initial cancer lesion is 196 from breast (47%), 90 from lung (21 %), 18 from gastrointestinal tract (4%), 9 from kidney (2%), 9 from skin (2%), 9 from prostate (2%), and 16 from other sites (4%). The technologies we applied today such as CT and MRI are time-consuming and expensive with limited sensitivity and specificity. Therefore, early diagnosis of ocular metastasis for CRC patients is not an easy job but rather important. Serum tumor markers detection has the priority of repeatability, non-invasive and low cost. So far, there has been studies about the risk factors of different metastatic sites of CRC, which indicated the possibility of some biology markers to predict metastases [Table 5]. Those changes of bio-markers offered a potential method for clinical diagnosis and prediction. Thus, we analyzed the clinical features of OM and NOM patients and clarify the risk factors for ocular metastasis from CRC.

After analyzing the 1735 CRC patients' clinical data, it shows that the occurrence of OM was 1.12%, and male were more likely to suffer CRC. There are no significant different between OM patients and NOM patients in gender, age, histopathological type, tumor classification and tumor differentiation. And liver, lung and peritoneum were three most vulnerable metastasis sites. We detected four acknowledged serum tumor markers and found that CEA level has nonnegligible different between two groups, which was 96.34±168.00 ng/ml and 10.69±49.43 ng/ml respectively. The nonparametric sum up test showed that the difference was meaningful.

CEA is a glycoprotein related to cell adhesion, which is produced by the gastrointestinal tract during fetal development 23. It is a cell surface glycoprotein and the key function ligand for the metastasis of colon carcinoma 24. Currently the level of serum CEA has been recommended by the “National Institute of Clinical Excellence European Group on Tumor Markers” and the “American Society of Clinical Oncology” for observation after curative resection of CRC 25. CEA is significant in 70% of cases with the diagnosis of CRC, and is the most widely used CRC marker 11. CEA and CA-199 are late-stage markers of carcinogenesis, and serum concentrations are significantly elevated in metastatic colon cancer. Patients with older age have significantly elevated levels of both markers 18. Serum CEA level and CA19-9 level are risk factors for metastases in colorectal cancer such as liver 19, lungs, lymph nodes 18 and peritoneal 20. The serum levels of CEA was closely related with overall survival 21. A report in 1976 showed a markable increase in serum CEA levels in patients with ocular endodermal-derived metastatic lesions, in accord with metastatic disease and colorectal carcinoma 22.

Considering serum CEA were known as the specific tumor biomarker for metastasis of colorectal cancer, in order to enable its specificity to predict ocular metastasis in our study, we made comparison between OM patients and NOM patients with other organ metastases. The resulted noted that the discrepancy was statistically significant (P<0.001), which meant the elevated CEA level had its value to predicting ocular metastasis.

The result of our study indicated that CEA can be recognized as a risk factor for OM from CRC. And the cutoff value was 12.45 ng/ml with a sensitivity of 100% for diagnosing ocular metastasis in this study, and its specificity was 87.7%. The AUC of ROC curve was 0.943, which showed a relatively high accuracy in distinguish OM patients with colorectal cancer, and further revealed the excellent diagnostic value of CEA in predicting OM. The examination of serum CEA is economical, simple and fast. Moreover, it is of excellent sensitivity and specificity.

Although the results were significant, our study is limited to some extent. First of all, as a retrospective study, some patients were excluded from the study and it may lead to the deviation in analysis. Secondly, the concentration of tumor markers in serum was collected at the time of diagnosis, which lack of changes in line with tumors' progress. Third, the size of OM groups was small in this study comparing to the whole amount, which made the outcome not so convincing. But our study still implied that high concentration CEA in plasma is risky for CRC patients in developing OM. Finally, all the patients were all collected in the same hospital, which may present the selective bias. Thus, it is necessary to verify the results of this study by future prospective, a large sample size and multiple centers analysis. In order to validate our finding, we will set about a cohort study in the future. We will make follow-up study to tract CRC patients with CEA concentration higher than 12.45 ng/ml, and further figure out the validity of CEA to predict ocular metastases.

In this study, we found that about 1.12% patients with CRC developed ocular metastasis. The concentration of serum CEA was potentially risk for OM in patients with CRC, and the value of CEA is 12.45 ng/ml. For newly diagnosed colorectal patients, if their serum CEA>12.45 ng/ml, further examination such as head CT, MRI, fundus photographs and selected fluorescein angiograms should be made. Taken together, we considered CEA as an independent risk factor and its valuable role for predicting ocular metastasis in colorectal cancer patients.

## Figures and Tables

**Figure 3:**
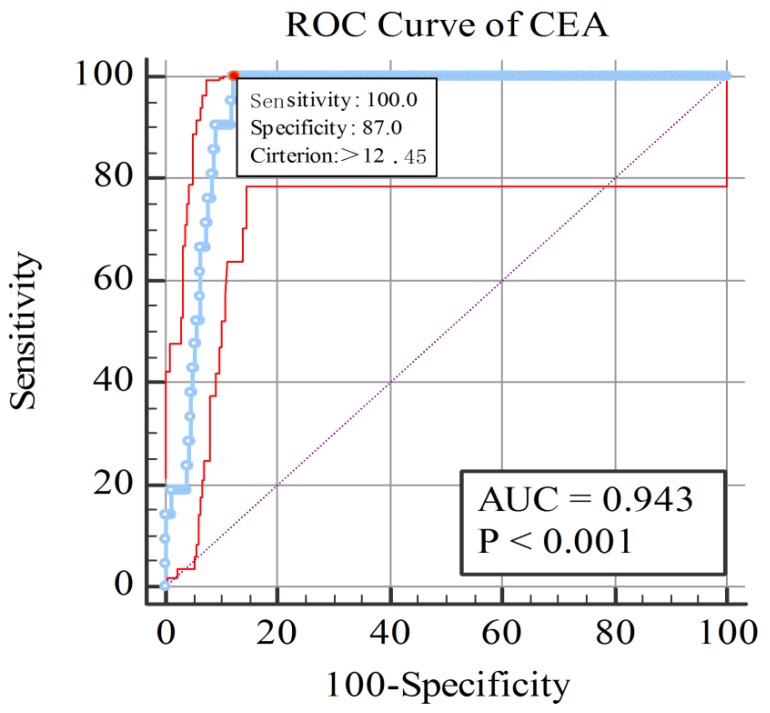
The receiver operating characteristics (ROC) curves of risk factor CEA for detecting OM in colorectal cancer. **Notes:** The area under the ROC curve were 0.934, (*p* < 0.001; 95% CI: 0.931-0.953) for CEA. **Abbreviations:** ROC, receiver operating characteristic; AUC, area under curve.

**Table 2:** The differences of clinical recognized serum tumor markers between patients with and without OM

Clinical features	OM group	NOM group	Z	P value*
AFP (ng/ml)	2.35±0.81	2.54±1.97	-0.395	0.693
CEA (ng/ml)	96.34±168.00	10.69±49.43	-6.988	< 0.001
CA-125 (μ/ml)	21.60±18.99	16.27±22.87	-1.766	0.077
CA-199(μ/ml)	8.39±5.15	9.13±4.54	-0.833	0.377

**Notes:** *P value: Comparison between OM group and NOM metastases group by nonparametric sum up tests. P<0.05 represented statistical significant. **Abbreviations:** OM, ocular metastasis; NOM, non-ocular metastasis.

**Table 1:** The clinical characteristics of patients with colorectal cancer

Patient characteristics	OM group(%)	NOM group(%)	Total number of patients(%) (n=1735)	P value*
**Gender, n(%)#**				
Male	16(76.2)	1045(61.0)	1061(61.2)	0.155
Female	5(23.8)	669(39.0)	674(38.8)	
**Age(years) #**				
<60	11(52.4)	857(50.0)	868(50.0)	0.828
≥60	10(47.6)	857(50.0)	867(50.0)	
Mean##	57.62±12.10	57.95±13.36	57.94±13.34	0.831
Range	15-95	35-82	15-95	
**Histopathological type, n(%)#**				
Adenocarcinoma	15(71.4)	1409(82.2)	1424(82.1)	0.459
Mucinous adenocarcinoma	2(9.5)	133(7.8)	135(7.8)	
Signet-ring cell carcinoma	0(0.0)	12(0.7)	12(0.7)	
Unknown	4(19.1)	160(9.3)	164(9.4)	
**Tumor classification#**				
Colon cancer	9(42.9)	746(43.5)	755(43.5)	0.586
Rectal cancer	10(47.6)	887(51.8)	897(51.7)	
Unknown	2(9.5)	81(4.7)	83(4.8)	
**Degree of tumor differentiation#**				
High	0(0.0)	35(2.0)	35(2.0)	0.839
Intermediate	13(61.9)	1142(66.6)	1155(66.6)	
Low	1(4.8)	80(4.7)	81(4.7)	
Undetermined	0(0.0)	0(0.0)	0(0.0)	
Unknown	7(33.3)	457(26.7)	464(26.7)	
**Distant metastasis**				
Liver	9	83	92	
Lung	11	23	34	
Brain	3	1	4	
Pancreas	1	3	4	
Pelvic	2	10	12	
Peritoneum	1	19	20	
Uterus, adnexa	1	5	6	
Stomach	0	1	1	

**Notes:** OM group included 16 orbital metastasis cases and 5 intraocular metastasis cases.

**Table 3:** The differences of serum CEA concentration between OM patients and NOM patients with other organ metastases

Clinical features	OM group(n=21)	NOM group(n=138)	Z	P value*
CEA (ng/ml)	96.34±168.00	22.59±89.92	-5.489	< 0.001

**Notes:** *P value: Comparison between OM group and NOM metastases group by nonparametric sum up tests. P<0.05 represented statistical significant. **Abbreviations:** OM, ocular metastasis; NOM, non-ocular metastasis.

**Table 4:** The binary logistic regression results

Factors	B	OR	OR (95% CI)	P value
AFP	-0.031	0.970	0.717-1.311	0.842
CEA	0.005	1.005	1.002-1.008	< 0.001
CA-125	-0.004	0.996	0.980-1.012	0.621
CA-199	-0.024	0.977	0.888-1.074	0.627

**Notes:** P <0.05 represented statistical significant **Abbreviations:** B, coefficient of regression; OR, odds ratio; CI, confidence interval.

**Table 5:** The risk factors of metastases of colorectal cancer

Author	Year	Metastatic sites	Risk factor
Yokomizo H et al^26^	2003	liver	Fas L
Qian LYet al^27^	2012	liver	CEA,VEGF,EGFR
Zhang D et al^19^	2013	liver	CEA,CA-199,CA-125
Pan HD et al^28^	2017	liver	PDGFAA
Saigusa S et al^29^	2013	liver, lung	DUSP4
Liu F et al^20^	2011	peritoneal	CEA, CA-199
Liu YL et al^30^	2011	lymph node	LDL-C

## References

[1] Roncucci L, Mariani F (2015). Prevention of colorectal cancer: How many tools do we have in our basket?. Eur J Intern Med.

[2] Siegel R, DeSantis C, Jemal A (2014). "Colorectal cancer statistics, 2014". CA Cancer J Clin.

[3] World Cancer Research Fund/American Institute for Cancer Research (2007). Food, Nutrition, Physical Activity, and the Prevention of Cancer: A Global Perspective. Washington DC: AICR.

[4] Jemal A, Bray F, Center MM (2011). Global cancer statistics. CA Cancer J Clin.

[5] Hess KR, Varadhachary GR, Taylor SH (2006). Metastatic patterns in adenocarcinoma. Cancer.

[6] Jardel P, Sauerwein W, Olivier T (2014). Management of choroidal metastases. Cancer Treat Rev.

[7] Georgalas I, Paraskevopoulos T, Koutsandrea C (2015). Ophthalmic Metastasis of Breast Cancer and Ocular Side Effects from Breast Cancer Treatment and Management: Mini Review.

[8] Zhong W, Yu Z, Zhan J (2015). Association of serum levels of CEA, CA199, CA125, CYFRA21-1 and CA72-4 and disease characteristics in colorectal cancer. Pathol Oncol Res.

[9] Liu X, Cai H, Wang Y (2012). Prognostic significance of tumour markers in chinese patients with gastric cancer.

[10] Zora Vukobrat-Bijedic, Azra Husic-Selimovic, Amela Sofic (2013). Cancer Antigens (CEA and CA 19-9) as Markers of Advanced Stage of Colorectal Carcinoma. Med Arch.

[11] McKeown E, Nelson DW, Johnson EK (2014). Current approaches and challenges for monitoring treatment response in colon and rectal cancer. J Cancer.

[12] Michelson JB, Felberg NT, Shields JA (1976). Carcinoembryonic antigen. Its role in the evaluation of intraocular malignant tumors. Arch Ophthalmol.

[13] Torre LA, Bray F, Siegel RL (2015). Global cancer statistics, 2012. CA Cancer J Clin.

[14] Daniele V (2017). F. Tauriello, Alexandre Calon, Enza Lonardo, et al. Determinants of metastatic competency in colorectal cancer. Mol Oncol.

[15] Linares P, Castanon C, Vivas S (2004). Bilateral choroidal metastasis as the initial manifestation of a rectal cancer. J Gastroenterol Hepatol.

[16] Kuo IC, Haller JA, Maffrand R (2008). Regression of a subfoveal choroidal metastasis of colorectal carcinoma after intravitreous bevacizumab treatment.

[17] Shields CL, Shields JA, Gross NE (1997). Survey of 520 eyes with uveal metastases. Ophthalmology.

[18] Vukobrat-Bijedic Z, Husic-Selimovic A, Sofic A (2013). Cancer Antigens (CEA and CA 19-9) as Markers of Advanced Stage of Colorectal Carcinoma. Med Arch.

[19] Zhang D, Yu M, Xu T (2013). Predictive value of serum CEA, CA19-9 and CA125 in diagnosis of colorectal liver metastasis in Chinese population. Hepatogastroenterology.

[20] Liu F, Yu J, Liang YZ (2011). Associated risk factors of peritoneal metastasis in colorectal cancer. Zhonghua Wei Chang Wai Ke Za Zhi.

[21] Zhong W, Yu Z, Zhan J (2015). Association of serum levels of CEA, CA199, CA125, CYFRA21-1 and CA72-4 and disease characteristics in colorectal cancer. Pathol Oncol Res.

[22] Michelson JB, Felberg NT, Shields JA (1976). Carcinoembryonic antigen. Its role in the evaluation of intraocular malignant tumors. Arch Ophthalmol.

[23] Flamini E, Mercatali L, Nanni O (2006). Free DNA and carcinoembryonic antigen serum levels: an important combination for diagnosis of colorectal cancer. Clin. Cancer Res.

[24] Gold P (1965). and Freedman, S.O. Specific carcinoembryonic antigens of the human digestive system. J. Exp. Med.

[25] Duffy M.J (2001). Carcinoembryonic antigen as a marker for colorectal cancer: is it clinically useful? Clin. Chem.

[26] Yokomizo H, Yoshimatsu K, Ishibashi K (2003). Fas ligand expression is a risk factor for liver metastasis in colorectal cancer with venous invasion. Anticancer Res.

[27] Qian LY, Li P, Li XR, Chen DJ (2012). Multivariate analysis of molecular indicators for postoperative liver metastasis in colorectal cancer cases. Asian Pac J Cancer Prev.

[28] Pan HD, Peng YF, Xiao G1 (2017). High levels of serum platelet-derived growth factor-AA and human epidermal growth factor receptor-2 are predictors of colorectal cancer liver metastasis. World J Gastroenterol.

[29] Saigusa S, Inoue Y, Tanaka K (2013). Decreased expression of DUSP4 is associated with liver and lung metastases in colorectal cancer. Med Oncol.

[30] Liu YL, Qian HX, Qin L (2011). Serum LDL-C and LDL-C/HDL-C ratio are positively correlated to lymph node stages in males with colorectal cancer. Hepatogastroenterology.

